# A Low Geriatric Nutrition Risk Index Is Associated with Progression to Dialysis in Patients with Chronic Kidney Disease

**DOI:** 10.3390/nu9111228

**Published:** 2017-11-09

**Authors:** I-Ching Kuo, Jiun-Chi Huang, Pei-Yu Wu, Szu-Chia Chen, Jer-Ming Chang, Hung-Chun Chen

**Affiliations:** 1 Graduate Institute of Clinical Medicine, College of Medicine, Kaohsiung Medical University, Kaohsiung 807, Taiwan; peyto26@hotmail.com (I-C.K.); karajan77@gmail.com (J.-C.H.); wpuw17@gmail.com (P.-Y.W.); 2 Division of Nephrology, Department of Internal Medicine, Kaohsiung Medical University Hospital, Kaohsiung Medical University, Kaohsiung 807, Taiwan; jemich@kmu.edu.tw (J.-M.C.); chenhc@kmu.edu.tw (H.-C.C.); 3 Department of Internal Medicine, Kaohsiung Municipal Ta-Tung Hospital, Kaohsiung Medical University, Kaohsiung 801, Taiwan; 4 Department of Internal Medicine, Kaohsiung Municipal Hsiao-Kang Hospital, Kaohsiung Medical University, Kaohsiung 812, Taiwan; 5 Faculty of Medicine, College of Medicine, Kaohsiung Medical University, Kaohsiung 807, Taiwan

**Keywords:** geriatric nutritional risk index (GNRI), chronic kidney disease (CKD), progression to dialysis, echocardiographic parameters

## Abstract

Evaluating nutritional status is crucial to detecting malnutrition in patients with chronic kidney disease (CKD). The Geriatric Nutritional Risk Index (GNRI) has been associated with overall and cardiovascular mortality in the dialysis population. The aim of this study was to evaluate whether the GNRI is associated with progression to dialysis in patients with moderate to advanced CKD. We enrolled 496 patients with stage 3–5 CKD who had received echocardiographic examinations, and categorized them according to baseline GNRI values calculated using the serum albumin level and body weight. The renal end-point was defined as the commencement of dialysis. During follow-up (mean, 25.2 ± 12.5 months; range, 3.3–50.1 months), 106 (21.4%) of the patients progressed to dialysis. The GNRI was positively correlated with the left ventricular ejection fraction (LVEF) (*r* = 0.111, *p* = 0.014), and negatively correlated with the left ventricular mass index (*r* = −0.116, *p* = 0.001), left ventricular hypertrophy (*r* = −0.095, *p* = 0.035), and LVEF < 50% (*r* = −0.138, *p* = 0.002). In multivariable Cox analysis, a low GNRI, female sex, high systolic blood pressure, high fasting glucose, and low estimated glomerular filtration rate were independently associated with progression to dialysis. A low GNRI was independently associated with progression to dialysis in our study cohort. The GNRI may be useful in predicting the risk of adverse renal outcomes in patients with CKD stages 3–5. Additional studies are needed to explore whether an improvement in GNRI delays CKD progression.

## 1. Introduction

Nutritional management is important for patients with chronic kidney disease (CKD). The degree of malnutrition increases as CKD progresses and is closely associated with major adverse clinical outcomes, thereby resulting in increased rates of hospitalization and mortality [1]. Established reliable screening tools for nutritional status include the subjective global assessment (SGA) [2] and malnutrition-inflammation score (MIS) [3], both of which are subjective assessments. In addition, simplified objective assessment tools have also been developed including the Mini Nutritional Assessment-Short Form (MNA-SF) [4], Nutrition Risk Score (NRS) [5], Malnutrition Universal Screening Tool (MUST) [6], Malnutrition Screening Tool (MST) [7], and Geriatric Nutritional Risk Index (GNRI) [8]. The GNRI is calculated using the serum albumin level and body weight, and it was originally introduced as a simple tool to assess the nutritional status of elderly hospitalized patients [8]. Among them, the MIS scoring systems proposed by Kalantar-Zadeh et al. are most employed in CKD or dialysis patients, which have been validated as a marker associated with poor outcomes [3,9,10]. Besides, Yamada et al. have compared various assessing tools (MNA-SF, NRS, MUST, MST, and GNRI) and found that GNRI had the highest accuracy to assess malnutrition based on the MIS value because of the largest area under the receiver operating characteristic curve [11].

The GNRI is calculated using serum albumin level and body weight, and it was originally introduced as a simple tool to assess the nutritional status of elderly hospitalized patients [8]. Besides, GNRI has been demonstrated closely associated with elevated C-reactive protein which also indicated inflammatory status [12,13]. Several studies have reported that the GNRI can also be used to predict overall [14,15] and cardiovascular mortality [13] in patients undergoing chronic dialysis. Furthermore, the GNRI has recently been associated with the severity of vascular calcification in patients with CKD not on dialysis [16]. However, the relationship between GNRI and end-stage renal disease (ESRD) outcomes has not been thoroughly investigated in patients with CKD.

Despite these findings, whether or not the GNRI is a strong predictor of adverse renal outcomes has never been investigated. Therefore, the aim of this study was to investigate whether the GNRI was independently associated with progression to dialysis in these patients.

## 2. Subjects and Methods 

### 2.1. Study Patients and Design

This study enrolled 496 patients with stage 3–5 CKD from the Outpatient Department of Internal Medicine at a regional hospital in southern Taiwan from January 2007 to May 2010. The patients were followed up for more than three months to confirm the presence of CKD, and they were classified into three groups based on the stage of CKD according to the National Kidney Foundation-Kidney Disease Outcomes Quality Initiative (K/DOQI) guidelines at entry to the study [17] as follows: stage 3, estimated glomerular filtration rate (eGFR) 30 to 59 mL/min/1.73 m^2^; stage 4, eGFR 15 to 29 mL/min/1.73 m^2^; and stage 5, eGFR < 15 mL/min/1.73 m^2^. The patients who had significant mitral valve disease and those on maintenance hemodialysis (HD) were excluded. The study protocol was approved by the Institutional Review Board of the hospital, and all patients provided informed consent to participate in this study.

### 2.2. Evaluation of Cardiac Structure and Function

Echocardiographic parameters were evaluated by two independent experienced cardiologists who were blinded to the patients’ clinical data using standard two-dimensional and M mode images (VIVID 7, General Electric Medical Systems, Horten, Norway), and included the left ventricular (LV) end-diastolic volume (LVEDV), LV end-systolic volume (LVESV), left atrial diameter (LAD), LV internal diameter in diastole (LVIDd), LV posterior wall thickness in diastole (LVPWTd), and interventricular septal wall thickness in diastole (IVSTd). LV systolic function was evaluated according to the LV ejection fraction (LVEF), with a cutoff of <50% based on previous studies of preserved systolic function [18,19]. LV mass (LVM) was calculated according to a modification of Devereux’s method as: LVM = 1.04 × [(IVSTd + LVIDd + LVPWTd)^3^ − LVIDd^3^] − 13.6 g [20]. The left ventricular mass index (LVMI) was calculated as the LVM/body surface area. Left ventricular hypertrophy (LVH) was defined as LVMI ≥ 134 and ≥ 110 g/m^2^ in the male and female patients, respectively, according to previously reported cut-off values [20]. Offline analysis was performed using EchoPAC software (GE Medical Systems). An average measurement from three consecutive beats was used for each variable.

### 2.3. GNRI Calculation

Based on the original nutritional risk index for elderly subjects [8], the GNRI was calculated from the baseline serum albumin level and body weight as follows: GNRI = [14.89 × albumin (g/dL)] + [41.7 × (body weight/ideal body weight)]. The body weight/ideal body weight was defined as 1 when the patient’s actual body weight exceeded the ideal body weight. In this study, the ideal body weight was calculated from the patient’s height and a body mass index (BMI) of 22, as previously reported [11], to calculate the GNRI. BMI was calculated as body weight/height squared (kg/m^2^).

### 2.4. Collection of Demographic, Medical, and Laboratory Data

All demographic data and relevant medical histories of the patients including age, sex, smoking history, and comorbidities were recorded from medical records. Blood and urine samples were collected from the patients after a 12-h fast within one month of enrollment. All laboratory examinations were performed using an automated analyzer (COBAS Integra 400, Roche Diagnostics GmbH, Mannheim, Germany). Serum creatinine levels were calculated using the compensated Jaffé method (kinetic alkaline picrate) [21], and eGFR was calculated according to the Modification of Diet in Renal Disease (MDRD) study [22]. Proteinuria was defined as a score of ≥1+ on a dipstick test for spot urine.

### 2.5. Definition of Renal End-Point

The renal end-point was defined as starting dialysis, as determined by the regulations of the National Health Insurance program for dialysis therapy based on laboratory data, nutritional status, and symptoms and signs of uremia. Of those who reached the renal end-point, eGFR data were recorded until the commencement of dialysis, and the other patients were followed up until February 2011.

### 2.6. Statistical Analysis

Descriptive statistics are presented as percentages, means ± standard deviations, or medians (25th–75th percentiles) for triglycerides. Differences between study groups were assessed using one-way analysis of variance (ANOVA) for continuous variables and the chi-square test for categorical variables. Pearson’s correlation analysis was used to evaluate associations between the echocardiographic parameters and the GNRI. Multivariable linear regression analysis was used to determine the factors for GNRI. Multivariable Cox proportional hazards analysis was used to evaluate associations between baseline variables and progression to dialysis, with the results expressed as hazard ratios (HRs) and corresponding 95% confidence intervals (CIs). Baseline variables that were statistically significant (*p* < 0.05) in univariable analysis were entered into a multivariable model 1: GNRI, gender, diabetes mellitus, hypertension, and systolic blood pressure (significant in univariable analysis), and a multivariable model 2: GNRI, fasting glucose, hemoglobin, eGFR, CaXP product, uric acid, proteinuria, LAD > 4.7 cm, and LVH (significant in univariable analysis). We analyzed the time to the renal end-points using Kaplan-Meier survival curves with the log-rank test among the tertiles of GNRI. A direct comparison between albumin, BMI, and GNRI was performed in multivariable models. Incremental model performance was assessed using a change in the χ^2^ value. A *p* value < 0.05 was considered to indicate statistical significance. All statistical analyses were performed using SPSS software for Windows version 19.0 (Statistical Product and Service Solutions (SPSS) Inc., Chicago, IL, USA).

## 3. Results

Of the 496 patients enrolled in this study, 63.5% were men and 36.5% were women, with a mean age of 66.3 ± 12.2 years. Table 1 shows comparisons of the characteristics among the patients classified by the GNRI tertile with cutoff values of <104.0, 104.0–111.5, and ≥111.5, respectively. The mean GNRI values of the tertiles were 95.4 ± 9.8, 108.3 ± 2.0, and 117.4 ± 5.9, respectively. Compared to the patients in tertile 1, those in tertile 3 tended to be younger, have a higher BMI, higher levels of albumin, triglycerides, and hemoglobin, higher eGFR, and lower prevalence of proteinuria. In addition, the patients in tertile 3 had a higher LVEF and lower prevalence of LVEF < 50%.

### 3.1. Correlation between GNRI and Echocardiographic Parameters

Table 2 shows the correlations between the GNRI and echocardiographic parameters. The GNRI was positively correlated with LVEF (*r* = 0.111, *p* = 0.014), and negatively correlated with LVMI (*r* = −0.116, *p* = 0.001), LVH (*r* = −0.095, *p* = 0.035), and LVEF < 50% (*r* = −0.138, *p* = 0.002). However, the GNRI was not correlated with LVEDV, LVESV, or LAD > 4.7 cm.

### 3.2. Determinants of GNRI

Table 3 shows the determinants of GNRI. Following univariable analysis, GNRI had a positive correlation with hypertension, coronary artery disease, triglyceride, hemoglobin, and eGFR, and a negative correlation with age, proteinuria, LVH and LVEF < 50%. In the multivariable analyses, old age (unstandardized coefficient β: −0.097, *p* = 0.017), coronary artery (unstandardized coefficient β: 3.912, *p* = 0.011), high triglyceride (unstandardized coefficient β: 6.672, *p* = 0.001), high hemoglobin (unstandardized coefficient β: 1.343, *p* < 0.001), and LVEF ≧ 50% (unstandardized coefficient β: −5.261, *p* = 0.016) were significantly associated with high GNRI.

### 3.3. Risk of Progression to Dialysis 

**Figure 1:**
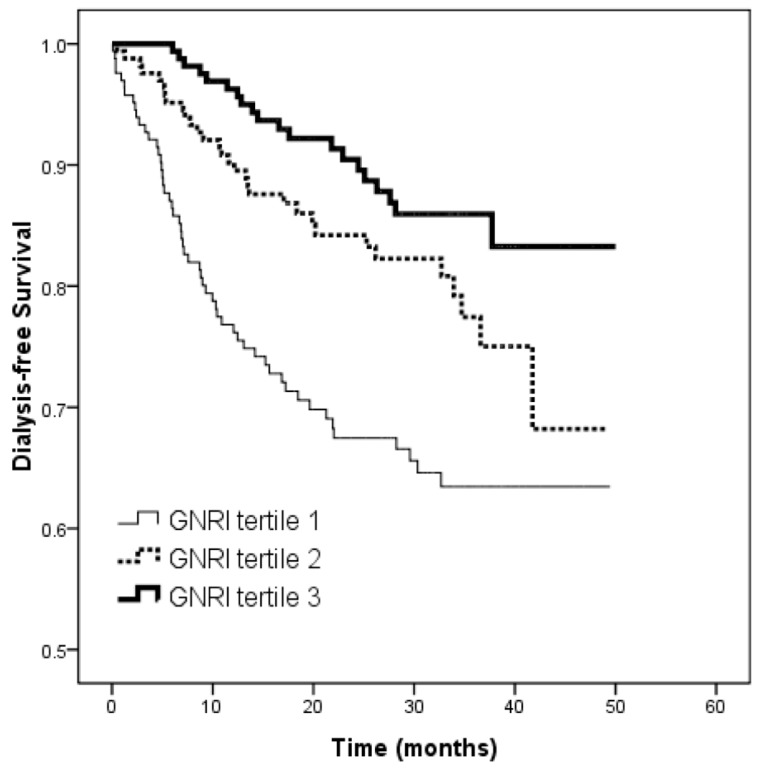
Kaplan-Meier analysis of dialysis-free survival according to tertiles of GNRI in patients with chronic kidney disease (log-rank *p* < 0.001). The group with the highest two tertiles had a better dialysis-free survival (all *p* < 0.05) than that with the lowest tertile of GNRI.

Over a mean follow-up period of 25.2 ± 12.5 months (range 3.3 to 50.1 months), 106 patients (21.4%) started dialysis. Table 4 shows the results of Cox proportional hazards regression analysis for the association between the GNRI to progression to dialysis. Univariable regression analysis showed that a lower GNRI (per 1 score; HR, 0.966; 95% CI, 0.958 to 0.977; *p* < 0.001), female sex, a history of diabetes and hypertension, high systolic blood pressure, high levels of fasting glucose, CaXP product and uric acid, low hemoglobin, low eGFR, proteinuria, LAD > 4.7 cm, and LVH were associated with a significantly higher risk of progression to dialysis. In the multivariable model, a lower GNRI (per 1 score; HR, 0.977; 95% CI, 0.964 to 0.990; *p* = 0.001), male sex, diabetes, high systolic blood pressure, low eGFR, and high CaXP product were independently associated with progression to dialysis. Table 5 demonstrates the multivariable analysis. In the multivariable model 1, a low GNRI (per 1 score; HR, 0.965; 95% CI, 0.955 to 0.976; *p* < 0.001), being female, and a high systolic blood pressure were independently associated with progression to dialysis. In the multivariable model 2, a low GNRI (per 1 score; HR, 0.975; 95% CI, 0.963 to 0.987; *p* < 0.001), high fasting glucose, low hemoglobin, and low eGFR were independently associated with progression to dialysis. Figure 1 illustrates the Kaplan-Meier curves for dialysis-free survival according to the tertiles of GNRI (log-rank *p* < 0.001). The unadjusted HR for tertile 2 versus quartile 1 was 0.509 (95% CI, 0.327 to 0.792, *p* = 0.003), compared to 0.310 (95% CI, 0.186 to 0.518, *p* < 0.001) for quartile 3 versus quartile 1. The patients in the highest two tertiles had a better dialysis-free survival than those in the lowest tertile of GNRI.

We further performed analysis using Lorentz’s formula, and still found that a low GNRI was still significantly associated with progression to dialysis in model 1 (per 1 score; HR, 0.947; 95% CI, 0.929 to 0.966; *p* < 0.001) and model 2 (per 1 score; HR, 0.959; 95% CI, 0.938 to 0.981; *p* < 0.001).

To avoid interaction with BMI, we further performed multivariable analysis after the exclusion of patients with BMI ≧ 30 kg/m^2^ (*n* = 61), and found a low GNRI was still significantly associated with progression to dialysis in model 1 (per 1 score; HR, 0.966; 95% CI, 0.954 to 0.978; *p* < 0.001) and model 2 (per 1 score; HR, 0.977; 95% CI, 0.963 to 0.991; *p* = 0.001). Similarly, after the exclusion of patients with BMI < 18.5 kg/m^2^ (*n* = 13), we still found that a low GNRI was significantly associated with progression to dialysis in model 1 (per 1 score; HR, 0.966; 95% CI, 0.955 to 0.978; *p* < 0.001) and model 2 (per 1 score; HR, 0.975; 95% CI, 0.962 to 0.987; *p* < 0.001).

### 3.4. Comparison of Albumin, BMI and GNRI in Progression to Dialysis

Because malnutrition may influence CKD progression, we further performed multivariable analysis in patients with albumin <3.5 g/dL (*n* = 50), and found that GNRI was significantly related to progression to dialysis (per 1 score; HR, 0.865; 95% CI, 0.798 to 0.938; *p* < 0.001) and outperformed albumin and BMI of the model. In a direct comparison, the multivariable model without albumin, BMI, and GNRI was not significantly improved by adding albumin (χ^2^ change = 3.197, *p* = 0.074) and BMI (χ^2^ change = 2.639, *p* = 0.104), respectively. Whereas adding GNRI resulted in significant improvement (χ^2^ change = 6.552, *p* = 0.010).

## 4. Discussion

In the present study, we found that a low GNRI was associated with LVH and low LVEF. Furthermore, a low GNRI was significantly associated with progression to dialysis in patients with moderate to severe CKD, independently of cardiac morphology and function.

Protein malnutrition and wasting have been reported to be common in patients with CKD [23], and they tend to develop and progress with a decline in kidney function. Many factors have been proposed to contribute to protein malnutrition and wasting, including an inadequate nutrient intake, acidemia, hormonal dysregulation, sustained inflammation, and changes in bowel flora [24], all of which can result in the progression of kidney disease and increased rates of morbidity and mortality. Thus, evaluating nutritional status plays a critical role in detecting malnutrition in patients with CKD. Several nutritional scoring systems have been proposed to assess nutritional status, including the SGA, MIS, and a modified version of the SGA, all of which require subjective assessments by the examiners [2,3]. Simplified screening tools using objective parameters have also been developed, including the MNA-SF [4], NRS [5], MUST [6], MST [7], and GNRI [8]. The GNRI is calculated using three common objective and definite measures (body weight, height, and serum albumin level) and it can easily be applied in clinical practice. Furthermore, the predictive value of the GNRI has been validated in both elderly hospitalized patients and patients undergoing chronic HD [8,11]. Of the aforementioned nutritional screening tools, the GNRI seems to be the most accurate in predicting malnutrition in HD patients when using the MIS as the reference standard [11]. The GNRI is a nutrition-related risk index, and as such, GNRI scores have been associated with nutritional status-related complications [8]. Several studies have also reported associations between the GNRI and adverse clinical outcomes in patients undergoing chronic HD [13,14,15,25], peritoneal dialysis [26], and in those with heart failure [27]. Previous cohort studies on maintenance HD patients have reported that the GNRI is associated with all-cause mortality and cardiovascular mortality [13,14,15]. Furthermore, another observational study also demonstrated that a low GNRI, particularly in combination with a high level of C-reactive protein, was correlated with more severe abdominal aortic calcification in patients with CKD [16]. Recently, Takahashi et. al investigated the predictive ability of nutritional status using diagnostic standards for protein energy wasting (PEW) according to the International Society of Renal Nutrition and Metabolism (ISRNM) criteria and GNRI. Their results indicated that the GNRI and ISRNM had comparable reliability to predict mortality among Japanese dialysis patients [28]. Taken together, these findings support that the GNRI could be used as a nutrition-related factor to predict morbidity and mortality. Besides, in our study, in a direct comparison, GNRI outperformed albumin and BMI in predicting progression to dialysis in CKD patients with albumin <3.5 g/dL. Furthermore, GNRI could add a significant incremental prognostic value beyond the conventional clinical and echocardiographic parameters in CKD patients with albumin <3.5 g/dL. To the best of our knowledge, this study is the first to report an association between the GNRI and ESRD as the renal end-point. Regression analysis showed that the GNRI independently predicted the risk of dialysis in patients with stages 3–5 CKD.

Defining an ideal cutoff value of the GNRI to definitively represent malnutrition is a challenge. Kobayashi et al. used a GNRI cutoff value for mortality in patients undergoing HD of 90 based on the highest positive likelihood and risk ratios [3], which is close to the cutoff value of 91.2 reported by Yamada et al. for the detection of malnutrition [11]. In addition, Takahashi el al. used a cut-off value of 92.2 as determined by receiver operating curve analysis to compare the predictability of PEW according to the ISRNM and GNRI for all-cause mortality in Japanese patients on HD [28]. We found that the patients in the lowest GNRI tertile (<104) had the highest risk of ESRD, and that this risk was higher than previously reported in HD patients. In patients with ESRD undergoing dialysis, in addition to an inadequate spontaneous nutrient intake, other protein catabolic and inflammatory stimuli have been associated with dialysis, thereby resulting in a higher risk of developing PEW [29]. These findings may suggest that our patients with poor outcomes had higher GNRI values then those on HD, since our patients had stages 3–5 CKD without dialysis.

Previous studies have suggested that persistent concentric and eccentric LVH predisposes patients with CKD to higher risks of ESRD, cardiovascular events, and mortality independently of LV geometry [30,31,32,33,34]. Furthermore, we previously reported that patients with a low BMI and a higher LVMI had a significantly higher risk of cardiovascular events compared to those with a higher BMI [30]. This phenomenon is thought to involve “reverse epidemiology” in patients with CKD. In addition, Maruyama et al. reported that the presence of LVH in combination with a lower GNRI was associated with a higher risk of cardiovascular events in 161 patients, of whom only 25% had an eGFR < 60 mL/min/1.73 m^2^ [35]. An increased BMI in patients with CKD has also been reported to be independently associated with LVH in addition to other hemodynamic-promoting factors [36]. However, when taking malnutrition and “reverse epidemiology” into consideration, a lower BMI with a higher LVMI has been reported to result in poor cardiovascular outcomes in patients with stages 3–5 CKD [37]. In the present study, univariable regression analysis indicated that LVH was a predictor of ESRD; however, the significance disappeared after adjustments for multiple variables. In contrast, the GNRI maintained a significant association with progression to dialysis after adjustments. Although a higher BMI has been shown to be associated with LVH with non-hemodynamic effects [36,38], the GNRI as a nutritional marker may provide further information on LV remodeling relating to the clinical entities of malnutrition, inflammation, and atherosclerosis. We therefore hypothesize that the GNRI, as a nutritional indicator, may be more reliable than LVH in predicting dialysis in patients with stages 3–5 CKD.

Another important finding of this study is that a low GNRI was associated with a high LVMI, high prevalence of LVH, and low LVEF. The mechanism to explain the association between fluid status and nutritional status is unclear. Fluid overload can lead to gastrointestinal edema and poor ingestion [39]. In addition, fluid status can affect inflammatory status, and the subsequent inflammatory conditions can activate the ubiquitin-proteasome proteolytic system thereby resulting in the loss of muscle mass [40,41]. Furthermore, a better fluid status has been associated with an improved nutritional status, whereas a poor fluid status has been associated with the development of malnutrition [40]. In this study, we found that a low GNRI was associated with LVH and impaired LV systolic function, although the causality could not be identified.

There are several limitations to this study. First, we used baseline GNRI rather than mean GNRI. In addition, we did not obtain data of time-dependent changes in albumin and BMI, and we were thus not able to investigate the association between weight variation and outcomes. In addition, BMI is limited to differentiating fat and lean mass, and therefore we will use body composition analysis using dual-energy X-ray absorption to elucidate this issue in future studies. Second, we did not include inflammatory markers such as C-reactive protein into the analysis because of missing data. Chronic inflammation has been linked to malnutrition, and this is a potential confounder of the associations observed in this study. Third, BMI may be misleading in the presence of edema, which is common in patients with advanced CKD. Finally, GNRI was originally developed by modifying the nutritional risk index for elderly subjects. As Yamada et al. has reported, the ideal body weight was calculated from the patient’s height and a BMI of 22, which was a simpler equation than Lorentz’s original formula. He also demonstrated that there was only little difference between two formulas (*r* = 0.999, *p* < 0.001) [11].

In conclusion, in patients with stages 3–5 CKD, a low GNRI was an independent determinant of progression to dialysis. Using the GNRI to evaluate and manage nutritional status in patients with CKD may be important in predicting the commencement of dialysis. Additional studies are needed to explore whether an improvement in GNRI delays CKD progression.

## Figures and Tables

**Table 1:** Clinical characteristics of patients according to tertiles of GNRI.

Characteristics	Tertile 1 (*n* = 166)	Tertile 2 (*n* = 166)	Tertile 3 (*n* = 164)	*p*	All Patients (*n* = 496)
GNRI (score)	95.4 ± 9.8	108.3 ± 2.0 *	117.4 ± 5.9 *^,†^	<0.001	107.2 ± 11.4
Age (year)	68.0 ± 11.6	67.3 ± 11.8	63.6 ± 12.7 *^,†^	0.002	66.3 ± 12.2
Male gender (%)	62.7	62.7	65.2	0.854	63.5
Smoking history (%)	34.5	28.9	29.9	0.447	31.3
Diabetes mellitus (%)	54.2	54.8	59.1	0.618	56.0
Hypertension (%)	78.9	81.3	87.2	0.127	82.5
Coronary artery disease (%)	6.6	12.7	14.6	0.057	11.3
Cerebrovascular disease (%)	16.3	15.7	13.4	0.750	15.1
Systolic blood pressure (mmHg)	141.7 ± 25.5	140.2 ± 19.5	141.9 ± 18.0	0.729	141.3 ± 21.2
Diastolic blood pressure (mmHg)	77.9 ± 13.6	78.3 ± 11.7	81.3 ± 12.8	0.031	79.2 ± 12.8
Body mass index (kg/m^2^)	21.9 ± 2.7	25.2 ± 2.3 *	28.9 ± 3.2 *^,†^	<0.001	25.3 ± 4.0
**Laboratory parameters**					
Albumin (g/dL)	3.73 ± 0.44	4.07 ± 0.28 *	4.24 ± 0.26 *^,†^	<0.001	4.01 ± 0.40
Fasting glucose (mg/dL)	121.0 ± 52.3	126.5 ± 60.2	131.6 ± 62.4	0.259	126.4 ± 58.5
Triglyceride (mg/dL)	116 (82–177)	134.5 (103–199.8)	159 (107–225) *	<0.001	137.5 (96–201)
Total cholesterol (mg/dL)	192.0 ± 51.7	195.3 ± 45.2	194.5 ± 45.6	0.812	194.0 ± 47.5
Hemoglobin (g/dL)	10.6 ± 2.2	11.7 ± 2.2 *	12.6 ± 2.1 *^,†^	<0.001	11.6 ± 2.3
eGFR (mL/min/1.73 m^2^)	21.9 ± 13.5	26.7 ± 14.1 *	29.7 ± 14.0 *	<0.001	26.1 ± 14.2
CaXP product (mg^2^/dL^2^)	39.2 ± 8.5	38.0 ± 9.5	97.9 ± 8.0	0.332	38.3 ± 8.7
Uric acid (mg/dL)	8.0 ± 2.6	8.2 ± 2.0	8.4 ± 2.1	0.161	8.2 ± 2.2
Proteinuria (%)	75.8	61.2 *	61.6 *	0.006	66.2
**Echocardiographic data**					
LVEDV (mL)	112.6 ± 39.9	114.7 ± 38.1	119.4 ± 40.4	0.279	115.6 ± 39.5
LVESV (mL)	39.2 ± 26.5	38.1 ± 20.9	37.9 ± 27.8	0.875	38.4 ± 25.2
LAD > 4.7 cm (%)	6.0	7.2	6.1	0.883	6.5
LVMI (g/m^2^)	150.2 ± 56.1	135.3 ± 45.7 *	140.2 ± 47.3	0.022	141.9 ± 50.2
LVH (%)	65.7	55.4	56.7	0.118	59.3
LVEF (%)	67.0 ± 12.8	68.3 ± 9.7	69.7 ± 9.5 *	0.081	68.3 ± 10.8
LVEF < 50% (%)	10.2	4.2	2.4 *	0.005	5.6

Abbreviations: GNRI, geriatric nutrition risk index; eGFR, estimated glomerular filtration rate; CaXP product, Calcium-phosphorous product; LVEDV, left ventricular end-diastolic volume; LVESV, left ventricular end-systolic volume; LAD, left atrial diameter; LVMI, left ventricular mass index; LVH, left ventricular hypertrophy; LVM, left ventricular mass; LVEF, left ventricular ejection fraction. The cutoff values of tertiles of GNRI were <104.0, 104.0–111.5, ≧111.5, respectively. * *p* < 0.05 compared with tertile 1; ^†^ *p* < 0.05 compared with tertile 2.

**Table 2:** Correlation between GNRI and echocardiographic parameters in study patients.

Echocardiographic Parameters	Pearson’s *r*	*p*
LVEDV (mL)	0.033	0.463
LVESV (mL)	−0.049	0.275
LAD > 4.7 cm (%)	−0.007	0.874
LVMI (g/m^2^)	−0.116	0.001
LVH (%)	−0.095	0.035
LVEF (%)	0.111	0.014
LVEF < 50% (%)	−0.138	0.002

Values expressed as *r*. Abbreviations are the same as in Table 1.

**Table 3:** Determinants of GNRI in study patients.

Parameter	Univariable		Multivariable	
	Unstandardized Coefficient β (95% CI)	*p*	Unstandardized Coefficient β	*p*
Age (per 1 year)	−0.137 (−0.219, −0.055)	0.001	−0.097 (−0.176, −0.018)	0.017
Male versus female	−0.508 (−2.600, 1.583)	0.633	–	–
Smoking history	−1.174 (−3.344, 0.997)	0.289	–	–
Diabetes mellitus	0.599 (−1.430, 2.628)	0.562	–	–
Hypertension	2.666 (0.028, 5.304)	0.048	1.898 (−0.689, 4.486)	0.150
Coronary artery disease	4.066 (0.904, 7.229)	0.012	3.912 (0.919, 6.905)	0.011
Cerebrovascular disease	−1.318 (−4.127, 1.491)	0.357	–	–
Systolic blood pressure (per 1 mmHg)	−0.013 (−0.062, 0.037)	0.502	–	–
Diastolic blood pressure (per 1 mmHg)	0.076 (−0.005, 0.157)	0.065	–	–
Laboratory parameters				
Fasting glucose (per 1 mg/dL)	0.007 (−0.011, 0.024)	0.435	–	–
Triglyceride (per log 1 mg/dL)	9.154 (5.061, 13.248)	<0.001	6.672 (2.687, 10.657)	0.001
Total cholesterol (per 1 mg/dL)	0.013 (−0.009, 0.034)	0.242	–	–
Hemoglobin (per 1 g/dL)	1.548 (1.141, 1.956)	<0.001	1.343 (0.775, 1.910)	<0.001
eGFR (per 1 mL/min/1.73 m^2^)	0.165 (0.096, 0.235)	<0.001	0.003 (−0.101, 0.107)	0.954
CaXP product (per 1 mg^2^/dL^2^)	−0.089 (−0.207, 0.208)	0.136	–	–
Uric acid (per 1 mg/dL)	0.197 (−0.253, 0.648)	0.390	–	–
Proteinuria	−2.578 (−4.702, −0.455)	0.017	−1.037 (−3.506, 1.432)	0.409
Echocardiographic data				
LVEDV (per 1 mL)	0.010 (−0.016, 0.035)	0.463	–	–
LVESV (per 1 mL)	−0.022 (−0.062, 0.018)	0.275	–	–
LAD > 4.7 cm	−0.332 (−4.432, 3.768)	0.874	–	–
LVH	−2.199 (−4.239, −0.158)	0.035	−0.345 (−2.392, 1.703)	0.741
LVEF < 50%	−0.832 (−11.154, −2.509)	0.002	−5.261 (−9.531, −0.991)	0.016

Values expressed as unstandardized coefficient β and 95% confidence interval (CI). Abbreviations are same as Table 1.

**Table 4:** Risk factors of progression to dialysis using the univariable Cox proportional hazards model.

Parameter	Univariable	
	HR (95% CI)	*p*
GNRI (per 1 score)	0.966 (0.958–0.977)	<0.001
Age (per 1 year)	0.988 (0.973–1.004)	0.137
Male versus female	0.520 (0.354–0.762)	0.001
Smoking (ever versus never)	1.049 (0.695–1.585)	0.819
Diabetes mellitus	1.796 (1.193–2.704)	0.005
Hypertension	2.863 (1.392–5.888)	0.004
Coronary artery disease	1.413 (0.818–2.441)	0.215
Cerebrovascular disease	1.255 (0.755–2.086)	0.381
Systolic blood pressure (per 1 mmHg)	1.025 (1.017–1.033)	<0.001
Diastolic blood pressure (per 1 mmHg)	0.998 (0.983–1.013)	0.793
Laboratory parameters		
Fasting glucose (per 1 mg/dL)	1.003 (1.001–1.006)	0.017
Triglyceride (per log 1 mg/dL)	1.017 (0.453–2.284)	0.968
Cholesterol (per 1 mg/dL)	1.001 (0.997–1.006)	0.512
Hemoglobin (per 1 g/dL)	0.595 (0.539–0.657)	<0.001
eGFR (per 1 mL/min/1.73 m^2^)	0.844 (0.817–0.872)	<0.001
CaXP product (per 1 mg^2^/dL^2^)	1.070 (1.056–1.085)	<0.001
Uric acid (per 1 mg/dL)	1.106 (1.011–1.213)	0.028
Proteinuria	15.015 (5.527–40.787)	<0.001
LAD > 4.7 cm	2.298 (1.285–4.109)	0.005
LVH	4.234 (2.486–7.210)	<0.001
LVEF < 50%	1.532 (0.744–3.157)	0.247

Values express as hazard ratios (HR) and 95% confidence interval (CI). Abbreviations are the same as in Table 1.

**Table 5:** Risk factors of progression to dialysis using the multivariable Cox proportional hazards model.

Parameter	Multivariable: Model 1		Multivariable: Model 2	
	HR (95% CI)	*p*	HR (95% CI)	*p*
GNRI (per 1 score)	0.965 (0.955–0.976)	<0.001	0.975 (0.963–0.987)	<0.001
Male versus female	0.565 (0.383–0.832)	0.004		
Diabetes mellitus	1.508 (0.988–2.304)	0.057		
Hypertension	1.986 (0.951–4.149)	0.068		
Systolic blood pressure (per 1 mmHg)	1.021 (1.012–1.029)	<0.001		
Laboratory parameters				
Fasting glucose (per 1 mg/dL)			1.003 (1.001–1.006)	0.017
Hemoglobin (per 1 g/dL)			0.885 (0.770–1.018)	0.087
eGFR (per 1 mL/min/1.73 m^2^)			0.875 (0.838–0.913)	<0.001
CaXP product (per 1 mg^2^/dL^2^)			1.018 (0.996–1.040)	0.111
Uric acid (per 1 mg/dL)			1.046 (0.934–1.171)	0.436
Proteinuria			2.991 (0.702–12.750)	0.139
LAD > 4.7 cm			1.412 (0.741–2.691)	0.294
LVH			1.583 (0.914–2.743)	0.101

Values express as hazard ratios (HR) and 95% confidence interval (CI). Abbreviations are the same as in Table 1. Covariates in the model 1 included GNRI, gender, diabetes mellitus, hypertension and systolic blood pressure (significant in univariable analysis). Covariates in the model 2 included GNRI, fasting glucose, hemoglobin, eGFR, CaXP product, uric acid, proteinuria, LAD > 4.7 cm, and LVH (significant in univariable analysis).
